# Draft Genome Sequence of the Dimorphic Yeast *Yarrowia lipolytica* Strain W29

**DOI:** 10.1128/genomeA.01211-15

**Published:** 2015-11-25

**Authors:** Kyle R. Pomraning, Scott E. Baker

**Affiliations:** Environmental Molecular Sciences Laboratory, Pacific Northwest National Laboratory, Richland, Washington, USA

## Abstract

Here, we present the draft genome sequence of the dimorphic ascomycete yeast *Yarrowia lipolytica* strain W29 (ATCC 20460). *Y. lipolytica* is a commonly employed model for the industrial production of lipases, small molecules, and more recently for its ability to accumulate lipids.

## GENOME ANNOUNCEMENT

We sequenced genomic DNA from six mutants derived from UV mutagenesis of *Yarrowia lipolytica* strain W29 (ATCC 20460) using a whole-genome shotgun sequencing approach on an Illumina HiSeq instrument. Ten million paired-end 150-bp reads with an average insert size of 243 ± 28 bp (standard deviation) from each strain (60 million reads total) were assembled into a consensus sequence of 369 contigs using Velvet version 1.2.10 (1). A *k*-mer length of 87 nucleotides (nt) was chosen to optimize for the highest *N*_50._ The resulting assembly had a size of 20.3 Mb (*N*_50_, 125,743 bp; *N*_max_, 356,851 bp; median coverage, 148.2×) and G+C content of 49.0%. The contigs were scaffolded to six chromosomes based on the reference strain CLIB122 (2) using CONTIGuator (3) and named YaliW29_A through YaliW29_F. Unscaffolded contigs include three high-coverage contigs that are predicted to be repetitive nuclear DNA. These are the 18S rRNA repeats (2,092 bp; ∼182 copies), the 26S rRNA repeats (2,283 bp; ∼177 copies), and a repeat (2,289 bp; ∼20 copies) with high homology to the non-long terminal repeat (LTR) retrotransposon Ylli (4). Fifteen short AT-rich contigs (44,803-bp total, 22.6 ± 1.7% G+C content [standard deviation]) with high coverage (24.0 ± 1.8× [standard deviation] that of the nuclear genome) were identified that aligned well to previously published *Y. lipolytica* mitochondrial sequence (5), against which they were scaffolded to generate YaliW29_M.

Strain W29 was isolated from wastewater in Paris, France, and was used in early inbreeding programs to develop *Y. lipolytica* as a model for genetic studies (6). This lineage has contributed to the genetic background of the widely used Po1 series of backcrossed auxotrophic strains (7, 8) that are employed by labs worldwide for genetic studies. The best annotated reference genome for *Y. lipolytica* was produced using strain CLIB122 (2). We mapped the W29-derived high-throughput sequencing reads to CLIB122 using Bowtie 2 (9) to identify differences between these strains. Analysis using SAMtools (10), Pindel (11), and custom Perl scripts identified 841 indels, 21,541 single-nucleotide polymorphisms (SNPs), and 20,206,880 bases conserved with CLIB122, giving an average SNP density between CLIB122 and W29 of 1,065 SNPs/Mb. Ninety-five percent of the predicted coding sequences from CLIB122 align well to the W29 assembly (>90% full-length identity), while 287 align along less than half their length or not at all. The strain W29 genome sequence will aid future genetic and genomic studies using *Y. lipolytica*.

### Nucleotide sequence accession numbers.

This whole-genome shotgun project has been deposited at DDBJ/ENA/GenBank under the accession no. LJBI00000000. The version described in this paper is the first version, LJBI00000000.1.
